# Neopterin as an Effect Modifier of the Cardiovascular Risk Predicted by Total Homocysteine: A Prospective 2‐Cohort Study

**DOI:** 10.1161/JAHA.117.006500

**Published:** 2017-11-02

**Authors:** Espen Ø. Bjørnestad, Robert A. Borsholm, Gard F. T. Svingen, Eva R. Pedersen, Reinhard Seifert, Øivind Midttun, Per M. Ueland, Grethe S. Tell, Kaare H. Bønaa, Ottar Nygård

**Affiliations:** ^1^ Faculty of Medicine University of Bergen Norway; ^2^ Department of Heart Disease Haukeland University Hospital Bergen Norway; ^3^ Bevital AS Bergen Norway; ^4^ Laboratory of Clinical Biochemistry Haukeland University Hospital Bergen Norway; ^5^ Department of Clinical Science University of Bergen Norway; ^6^ Department of Global Public Health and Primary Care University of Bergen Norway; ^7^ Department of Public Health and Nursing Norwegian University of Science and Technology Trondheim Norway; ^8^ KG Jebsen Centre for Diabetes Research Bergen Norway

**Keywords:** acute myocardial infarction, biomarker, epidemiology, inflammation, Coronary Artery Disease, Epidemiology, Inflammation, Oxidant Stress, Risk Factors

## Abstract

**Background:**

Plasma total homocysteine (tHcy) is related to plasma neopterin, an indicator of interferon‐γ‐mediated immune activation, and both biomarkers positively predict cardiovascular risk. We examined whether the association between tHcy and subsequent risk of acute myocardial infarction (AMI) was modified by systemic concentrations of neopterin and C‐reactive protein among patients with coronary heart disease.

**Methods and Results:**

By Cox modeling, we explored the association between tHcy and risk of AMI in 4164 patients with suspected stable angina pectoris. Subgroup analyses were performed according to median levels of neopterin and C‐reactive protein. A replication study was performed among 3749 patients with AMI at baseline. Median follow‐up was 7.3 and 8.3 years among patients with stable angina pectoris and AMI, respectively. tHcy and neopterin correlated in both cohorts (*r*_s_=0.34 and *r*_s_=0.30 among stable angina pectoris and AMI patients, respectively, both *P*<0.001). tHcy predicted AMI in both cohorts, independent of B‐vitamin treatment. However, significant risk associations were confined to patients with plasma neopterin above the median (hazard ratios [95% confidence interval] per 1‐SD increment of log‐transformed tHcy 1.38 [1.26–1.50] and 1.18 [1.10–1.26] among stable angina pectoris and AMI patients, respectively) (*P*_int_<0.005 in both cohorts). Further, adding information on the interaction between tHcy and neopterin improved model discrimination and reclassification. tHcy and C‐reactive protein were weakly related, and no effect modification was found by C‐reactive protein.

**Conclusions:**

Among patients with coronary heart disease, tHcy predicted risk of AMI only in subjects with concomitantly elevated plasma neopterin. Our results motivate further research on the relationship between homocysteine metabolism, cellular immune activation, and atherothrombosis.

**Clinical Perspective**

**What Is New?**

- In this long‐term prospective study based on 2 cohorts totaling almost 8000 patients with suspected or verified coronary heart disease, we observed positive correlations between total homocysteine and the immune marker neopterin.
- A positive risk relationship between total homocysteine and acute myocardial infarction was confined to patients with concomitantly elevated plasma neopterin.
- Information on the interaction between total homocysteine and plasma neopterin improved reclassification of patients.

**What Are the Clinical Implications?**

- Our findings suggest an interrelationship between cellular immune activation, homocysteine metabolism, and atherothrombosis.

Observational studies have consistently reported an association between increased plasma total homocysteine (tHcy) and risk of ischemic heart disease.^1^ However, tHcy lowering B‐vitamin therapy in secondary prevention trials did not reduce overall cardiovascular risk.^2^ Further, a recent meta‐analysis of Mendelian randomization studies did not support lifelong moderate hyperhomocysteinemia as a causal risk factor for coronary heart disease (CHD).^3^

Growing evidence indicates that plasma tHcy increases in parallel with immune activation.^4^ In particular, tHcy is closely correlated with plasma neopterin.^4^, ^5^, ^6^, ^7^ This pteridine is released from activated monocytes and macrophages upon stimulation with the Th1‐type cytokine interferon‐γ (IFN‐γ),^8^ and reflects the cellular immune response. Also, neopterin is a sensitive marker of reactive oxygen species generation in macrophages.^9^ Circulating neopterin is related to the presence of multiple complex atherosclerotic plaques in patients with CHD,^9^ and recent studies have shown that neopterin predicts future cardiovascular risk in subjects with stable angina pectoris,^10^, ^11^, ^12^ patients hospitalized for acute myocardial infarction (AMI),^13^ and community‐dwelling older adults without previous CHD.^14^

Persistent immune activation^15^ and accompanying oxidative stress^16^ may considerably influence homocysteine homeostasis, suggesting an interrelationship between tHcy, neopterin, and risk of cardiovascular disease. Notably, tHcy lowering B‐vitamin therapy had no effect on systemic concentrations of neopterin^17^ or other inflammatory markers.^5^ This finding raises the question of whether immune activation underlies the cardiovascular risk associated with elevated tHcy. However, to our knowledge, no data exist regarding cardiovascular risk prediction by tHcy according to inflammatory status.

The aim of this study was to explore whether the association between plasma tHcy and risk of AMI was modified by neopterin and CRP (C‐reactive protein) concentrations in a large cohort of patients undergoing coronary angiography for stable angina pectoris. Moreover, we sought to replicate the results in an independent high‐risk cohort of patients hospitalized for AMI.

## Methods

### Study Population

The present study comprised patients from 2 independent cohorts: the WECAC (Western Norway Coronary Angiography Cohort) 18 and the NORVIT (Norwegian Vitamin Trial),^19^ which both have been described in detail elsewhere. WECAC recruited 4164 patients who underwent elective coronary angiography because of suspected stable angina pectoris during 2000 to 2004. Of these patients, 2568 (61.7%) were enrolled in the WENBIT (Western Norway B‐vitamin intervention trial),^20^ a randomized, double‐blind, placebo‐controlled secondary prevention trial with B‐vitamins. In the current study, WECAC was the primary cohort.

NORVIT was a multicenter study enrolling 3749 subjects between 1998 and 2002. Patients were eligible for enrolment if they had been hospitalized for AMI within 7 days before randomization. The treatment design was identical to WENBIT.

The studies fulfilled the Declaration of Helsinki, and protocols were approved by the Regional Committee for Medical and Health Research Ethics in Western Norway. All participants provided written informed consent.

### Baseline Data and Biochemical Analyses

The collection of baseline data and biochemical analyses has previously been described in detail.^18^, ^19^ Information on medical history, medications, and risk factors was provided from self‐administered questionnaires, and subsequently checked against medical records when available. Smoking status was classified as “current” versus “never or former.” Hypertension was defined by pre‐existing diagnosis. Diabetes mellitus included types 1 and 2 and was defined by self‐reported diagnosis. In WECAC, patients were also classified as having diabetes mellitus if they had a baseline fasting plasma glucose >7 mmol/L or nonfasting plasma glucose >11.1 mmol/L.

Routine laboratory measurements were performed on fresh samples at each recruiting hospital. Blood samples for biobanking were frozen at −80°C until analyzed in 2007. Blood samples in the WECAC were immediately frozen, while samples in NORVIT were frozen within 48 hours after being shipped by mail. Serum CRP was measured using an ultrasensitive immunoassay, with a lower detection limit of 0.17 mg/L, applying the Behring nephelometer II system (coefficient of variation 8.1–11.4%; N Latex CRP mono; Behring Diagnostics, Marburg, Germany). Data on CRP were available in the WECAC only. Analyses on tHcy and neopterin were performed at Bevital AS, Bergen, Norway (www.bevital.no). Plasma tHcy was analyzed using a modified chromatography method based on ethylchloroformate derivatization (lower detection limit 0.1 μmol/L, within‐ and between‐day coefficients of variation 0.9–2.2% and 2.1–2.2%, respectively).^21^ Plasma neopterin was analyzed by liquid chromatography–tandem mass spectrometry (lower detection limit 0.7 nmol/L, within‐ and between‐day coefficients of variation 2.9–4.6% and 6.2–10.0%, respectively).^22^

### Follow‐Up and Clinical End Points

Information on study end points was collected from the CVDNOR (Cardiovascular Disease in Norway) project (http://www.cvdnor.no/),^23^ via linkage to each patient's 11‐digit personal identification number, unique to each Norwegian resident. The primary end point in the present study was new fatal or nonfatal MI (coded I21, I22, I46.1, R96, and R98 according to the *International Statistical Classification of Diseases 10th version*). The participants of both cohorts were followed from baseline until experiencing an AMI or through December 31, 2009.

### Statistical Analysis

Continuous variables are presented as medians (25th–75th percentile) and categorical variables as numbers (percentages). Patient baseline characteristics were assessed across tHcy quartiles; trends were tested by linear median regression for continuous variables and by binary and ordinal logistic regression for binary and ordinal variables, respectively. Baseline associations between plasma tHcy, plasma neopterin, and serum CRP were additionally explored by univariate and age‐ and sex‐adjusted Spearman rank correlations. Event‐free survival was estimated by the Kaplan–Meier method, and log‐rank tests were used to assess differences in survival across tHcy quartiles. Hazard ratios of AMI events per 1‐SD increment of log‐transformed tHcy were estimated with univariate, age‐ and sex‐adjusted and multivariate Cox regression analyses. The assumption of proportionality was tested by calculating Schoenfeld residuals and by visual examination of survival plots. The multivariate model included age (continuous), sex (binary), hypertension (binary), current smoking (binary), diabetes mellitus (binary), apolipoprotein (apo) B100 (continuous), and apoA1 (continuous). Potential effect modifications according to median plasma neopterin and serum CRP were assessed by including interaction terms to the regression models. Additionally, potential nonlinear interrelationships between tHcy, plasma neopterin, and risk of AMI were visualized by a surface spline from the unadjusted proportional hazards model.

Improvements in model discrimination were assessed by comparison of areas under the curve of receiver operating characteristics curves for the multivariate Cox models with and without the interaction terms, and tested by DeLongs test.^24^ Accordingly, logistic regression models containing the same covariates as the multivariate Cox model, with and without the interaction terms, were used to calculate the integrated discrimination improvement (IDI) and the continuous (category‐free) net reclassification improvement (>0), of which the latter indicates potential improvement in the reclassification of patients at risk.^25^

*P* values of <0.05 were considered to indicate statistical significance, and all statistical tests were 2‐tailed. For the statistical analyses, we used R version 3.3.2 (The R Foundation for Statistical Computing, Vienna, Austria, 2016); proportional hazard models with package “survival” version 2.40‐1; spline estimates with package “mgcv” version 1.8‐16; and 3D visualization with package “rgl” version 0.97.0.

## Results

### Baseline Characteristics (WECAC)

Baseline characteristics according to quartiles of plasma tHcy are presented in Table 1. Median (25th–75th percentile) plasma tHcy was 10.4 (8.7–12.6) μmol/L. The median (25th–75th percentile) age at inclusion was 62 (55–70) years, and 71.9% were male.

**Table 1:** Baseline Characteristics Among Participants of the WECAC (N=4164) According to Quartiles (n=1041) of Plasma tHcy

	Quartiles of Plasma tHcy				*P*_trend_
	First	Second	Third	Fourth	
Plasma tHcy, μmol/L	7.7 (6.9–8.2)	9.5 (9.1–10.0)	11.4 (10.9–12.0)	15.1 (13.6–17.6)	···
Male sex, n (%)	641 (61.7)	763 (73.4)	791 (76.1)	795 (76.5)	<0.001
Age, y	58 (51–65)	61 (54–68)	64 (56–71)	67 (59–74)	<0.001
Serum CRP, mg/L	1.6 (0.8–3.1)	1.7 (0.8–3.3)	1.8 (0.9–3.7)	2.2 (1.0–4.4)	<0.001
Plasma neopterin, nmol/L	7.3 (6.1–8.7)	7.7 (6.4–9.6)	8.5 (6.9–10.5)	9.9 (7.8–13)	<0.001
Current smoking, n (%)	259 (24.9)	264 (25.4)	252 (24.3)	298 (28.7)	0.096
Diabetes mellitus, n (%)	130 (12.5)	107 (10.3)	119 (11.5)	138 (13.3)	0.44
BMI, kg/m^2^	26.5 (24.3–29.1)	26.5 (24.3–28.7)	26.3 (24.2–29.0)	26.0 (23.8–28.7)	0.002
Hypertension, n (%)	432 (41.6)	444 (42.7)	482 (46.4)	585 (56.3)	<0.001
Extent of CAD, n (%)					<0.001
No significant stenosis	302 (29.1)	258 (24.8)	255 (24.5)	223 (21.5)	
1‐vessel disease	249 (24.0)	267 (25.7)	241 (23.2)	203 (19.5)	
2‐vessel disease	239 (23.0)	220 (21.2)	235 (22.6)	229 (22.0)	
3‐vessel disease	245 (23.6)	288 (27.7)	305 (29.4)	379 (36.5)	
Previous PCI, n (%)	355 (34.2)	364 (35.0)	339 (32.6)	312 (30.0)	0.023
Previous CABG, n (%)	212 (20.4)	215 (20.7)	217 (20.9)	232 (22.3)	0.292
LVEF, %	70 (60–70)	66 (60–70)	66 (60–70)	65 (56–70)	<0.001
Previous MI, n (%)	371 (35.7)	402 (38.7)	428 (41.2)	477 (45.9)	<0.001
eGFR, mL/min per 1.73 m^2^	98 (91–105)	93 (84–100)	88 (77–97)	78 (64–90)	<0.001
Serum lipids and apolipoproteins					
LDL‐C, mmol/L	2.9 (2.4–3.6)	2.9 (2.4–3.6)	3.0 (2.4–3.8)	2.9 (2.3–3.8)	0.034
HDL‐C, mmol/L	1.3 (1.0–1.5)	1.2 (1.0–1.5)	1.2 (1.0–1.5)	1.2 (1.0–1.5)	0.246
Total cholesterol, mmol/L	4.9 (4.3–5.7)	4.9 (4.3–5.6)	5.0 (4.3–5.8)	5.0 (4.2–5.8)	0.013
Triglycerides, mmol/L	1.5 (1.0–2.2)	1.5 (1.1–2.1)	1.5 (1.1–2.2)	1.5 (1.1–2.1)	1.000
ApoB100, g/L	0.9 (0.7–1.0)	0.9 (0.7–1.0)	0.9 (0.7–1.1)	0.9 (0.7–1.1)	0.007
ApoA1, g/L	1.3 (1.1–1.5)	1.3 (1.1–1.5)	1.3 (1.1–1.5)	1.3 (1.1–1.5)	0.030
Medications at discharge, n (%)					
Aspirin	833 (80.2)	854 (82.2)	855 (82.3)	851 (81.9)	0.324
Statins	830 (80.3)	848 (82.4)	821 (79.6)	824 (79.5)	0.367
β‐Blockers	715 (68.9)	776 (74.7)	778 (75.0)	741 (71.3)	0.235
ACEI	164 (15.8)	180 (17.3)	219 (21.1)	296 (28.5)	<0.001
Loop diuretics	58 (5.6)	65 (6.3)	113 (10.9)	216 (20.8)	<0.001

Continuous variables are presented as medians (25th–75th percentiles) and categorical variables as numbers (percentages). ACEI indicates angiotensin‐converting enzyme inhibitor; ApoA1, apolipoprotein A1; ApoB100, apolipoprotein B100; BMI, body mass index; CABG, coronary artery bypass grafting; CAD, coronary artery disease; CRP, C‐reactive protein; eGFR, estimated glomerular filtration rate; HDL‐C, high‐density lipoprotein cholesterol; LDL‐C, low‐density lipoprotein cholesterol; LVEF, left ventricular ejection fraction; MI, myocardial infarction; PCI, percutaneous coronary intervention; tHcy, total homocysteine; WECAC, Western Norway Coronary Angiography Cohort.

Subjects with higher plasma tHcy were more likely to have hypertension, previous AMI, and more extensive coronary artery disease at angiography. An inverse association was observed between tHcy with estimated glomerular filtration rate (eGFR) and left ventricular ejection fraction. tHcy was not associated with diabetes mellitus or current smoking.

Plasma tHcy showed a moderate positive correlation with plasma neopterin (univariate *r*_s_=0.34, adjusted *r*_s_=0.29, both *P*<0.001), which remained significant after adjustment for eGFR (*r*_s_=0.16, *P*<0.001). Both tHcy and neopterin were only weakly related to CRP (tHcy, univariate *r*_s_=0.11, adjusted *r*_s_=0.12; neopterin, univariate *r*_s_=0.19, adjusted *r*_s_=0.19, all *P*<0.001).

### Plasma tHcy and Risk of AMI (WECAC)

During median (25th–75th percentile) follow‐up of 7.3 (6.3–8.7) years, 638 (15.3%) patients experienced an AMI, of which 119 cases were fatal. Figure S1 depicts a Kaplan–Meier plot of event‐free survival time according to tHcy quartiles, showing decreased survival with increasing tHcy quartiles (*P*_log‐rank_<0.001). Univariate and multivariable hazard ratios (95% confidence interval [CI]) per 1‐SD increment of log‐transformed tHcy were 1.28 (1.20–1.37, *P*<0.001) and 1.17 (1.08–1.26, *P*<0.001), respectively. There were no statistically significant interactions according to WENBIT study treatment allocation (data not shown).

### tHcy‐AMI Risk Association According to Neopterin and CRP (WECAC)

**Figure 1:**
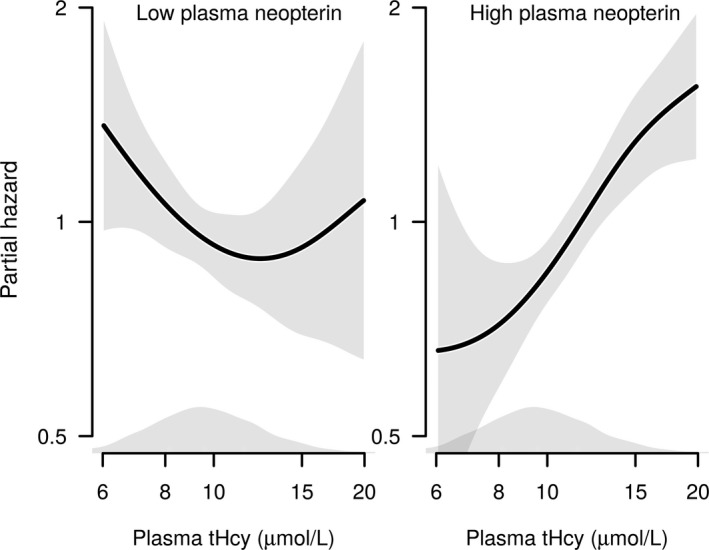
The age‐ and sex‐adjusted association between log‐transformed tHcy and risk of acute myocardial infarction in subgroups of low/high plasma neopterin (divided by median level). Shaded areas around the curves depict 95% confidence intervals. Kernel density plots show the distribution of tHcy. The *x*‐axis is trimmed, excluding the lower and upper 2.5 percentiles. tHcy indicates total homocysteine.

**Figure 2:**
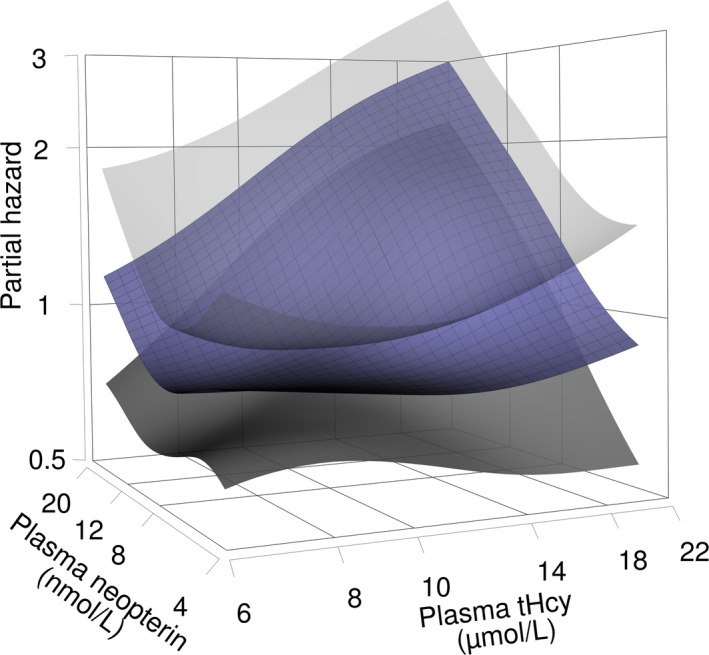
Surface plot depicting risk of acute myocardial infarction by log‐transformed concentrations of tHcy and plasma neopterin, estimated with a generalized additive model. The *x*‐ and *y*‐axis are trimmed, excluding the lower and upper 2.5 percentiles. Shaded areas around the surface plot depict 95% confidence intervals. tHcy indicates total homocysteine.

The relationship between tHcy and AMI according to plasma neopterin is shown in Figures 1 and 2. A positive risk relationship between tHcy and AMI was confined to patients with neopterin concentrations above the median (hazard ratio per 1‐SD increment of log‐transformed tHcy 1.38 [1.26–1.50], *P*<0.001), whereas no risk association was found among patients with neopterin concentrations below or at the median (hazard ratio per 1‐SD increment of log‐transformed tHcy 0.99 [0.88–1.11], *P*=0.89) (*P*_int_<0.001) (Table 2 and Figure 1). The effect modification tended to be stronger in patients not receiving statin therapy and patients without previous AMI (Tables S1 through S4). The risk association between tHcy and AMI was not modified by CRP (*P*_int_=0.35).

**Table 2:** Association Between Plasma tHcy and AMI According to Plasma Neopterin Among Participants in the WECAC

	Plasma Neopterin				*P*_int_
	≤Median^a^		>Median^a^		
	HR^b^	95% CI	HR^b^	95% CI	
Unadjusted	1.01	0.88 to 1.15	1.38	1.26 to 1.50	<0.001
Model 1^c^	0.95	0.83 to 1.10	1.29	1.18 to 1.43	<0.001
Model 2^d^	0.93	0.80 to 1.08	1.25	1.13 to 1.38	<0.001

AMI indicates acute myocardial infarction; CI, confidence interval; HR, hazard ratio; tHcy, total homocysteine; WECAC, Western Norway Coronary Angiography Cohort.

a Median: 8.2 nmol/L.

b Per 1‐SD increase in log‐transformed concentrations.

c Adjusted for age and sex.

d Adjusted for age, sex, diabetes mellitus, current smoking, hypertension, apolipoprotein A1, and apolipoprotein B.

### Model Discrimination and Reclassification (WECAC)

When added to the multivariate model, the interaction term between tHcy and neopterin significantly improved the receiver operating characteristics–areas under the curve (95% CI) (from 0.639 [0.613–0.664] to 0.650 [0.625–0.675], *P*=0.036) and the IDI (95% CI) (IDI 0.0079 [0.0046–0.0112], *P*<0.001). Accordingly, the tHcy‐neopterin interaction also improved reclassification of patients (net reclassification improvement >0 [95% CI]: 0.211 [0.120–0.302], *P*<0.001).

### Plasma tHcy, Neopterin, and Risk Prediction in an Independent Replication Cohort (NORVIT)

Baseline characteristics of the NORVIT cohort are presented in Table S5. Compared to WECAC, both plasma tHcy and neopterin were higher in NORVIT (both *P*<0.001). As in WECAC, tHcy and neopterin were positively correlated (univariate *r*_s_=0.30, adjusted *r*_s_=0.22, both *P*<0.001), also after adjustment for eGFR (*r*_s_=0.21, *P*<0.001).

During median (25th–75th percentile) follow‐up of 8.3 (2.4–9.8) years, 1341 (36%) persons experienced an AMI, of which 246 cases were fatal. Overall univariate and multivariable hazard ratios (95% CI) per 1‐SD increment of log‐transformed tHcy were 1.12 (1.06–1.18, *P*<0.001) and 1.06 (1.00–1.12, *P*=0.039), respectively. No statistically significant interactions were observed according to NORVIT study treatment allocation (data not shown).

Similar to the findings in WECAC, the association between plasma tHcy and risk of AMI was confined to subjects with neopterin levels above the median (*P*_int_=0.013) (Table 3). Adding the interaction term between tHcy and neopterin to the multivariate model improved reclassification of patients (net reclassification improvement >0 [95% CI]: 0.116 [0.043–0.185], *P*<0.001) and the IDI [95% CI] (IDI 0.0027 [0.0009–0.0044], *P*=0.003); however, the receiver operating characteristics–areas under the curve was not increased.

**Table 3:** Association Between Plasma tHcy and AMI According to Plasma Neopterin Among Participants of the Norwegian Vitamin Trial

	Plasma Neopterin				*P*_int_
	≤Median^a^		>Median^a^		
	HR^b^	95% CI	HR^b^	95% CI	
Unadjusted	0.99	0.91 to 1.09	1.18	1.10 to 1.26	0.004
Model 1^c^	0.98	0.90 to 1.07	1.09	1.01 to 1.17	0.013
Model 2^d^	1.00	0.91 to 1.10	1.08	1.00 to 1.17	0.045

AMI indicates acute myocardial infarction; BMI, body mass index; CI, confidence interval; HR, hazard ratio; tHcy, total homocysteine.

a Median: 8.8 nmol/L.

b Per 1‐SD increase in log‐transformed concentrations.

c Adjusted for age and sex.

d Adjusted for age, sex, diabetes mellitus, current smoking, and BMI.

## Discussion

### Principal Findings

In this prospective study of almost 8000 subjects in 2 independent cohorts of patients with suspected or verified CHD, tHcy was positively associated with the cellular immune activation marker neopterin. Further, tHcy predicted risk of AMI only in patients with concomitantly elevated neopterin concentrations. Adding the interaction term between tHcy and neopterin significantly improved reclassification of patients at risk. Our findings thus suggest an effect modification by cellular immune activation on the relationship between tHcy and atherothrombosis.

### Circulating tHcy and Inflammatory Markers Among CHD Patients

The observation of a significant correlation between tHcy and neopterin was consistent across the 2 cohorts, and agrees with previous findings among patients with vascular disease.^5^, ^6^, ^7^ tHcy and plasma neopterin are both associated with impaired renal function, which is a major cardiovascular disease risk factor.^26^ However, the positive relationships remained significant after controlling for eGFR, indicating additional mechanisms responsible for the parallel elevation of the 2 biomarkers.

Both tHcy and plasma neopterin were higher among patients with AMI than stable angina pectoris at baseline. Circulating neopterin has been observed to slightly increase during the first days following an AMI, possibly indicating increased monocyte/macrophage activation.^27^ In comparison, tHcy remains relatively stable during an acute coronary syndrome^28^; hence, increased tHcy is unlikely to be secondary to the coronary event per se, but may rather reflect the long‐term homocysteine status of these patients. Moreover, compared with patients with stable angina pectoris, those with AMI had higher age and lower eGFR, which could partly explain the elevated tHcy concentrations.^29^

At present, CRP is among the most studied inflammatory markers in cardiovascular risk assessment. In the current study, only weak correlations were found between CRP and neopterin. Also, we observed essentially no relationship between CRP and tHcy, in line with previous studies.^30^, ^31^ Accordingly, CRP did not modify the risk of AMI associated with increased tHcy, possibly reflecting that CRP represents a different inflammatory modality than neopterin, not closely linked to homocysteine homeostasis.

### tHcy, Cellular Immune Activation, and Redox Status

The lack of risk reduction by tHcy lowering B‐vitamin intervention in clinical trials^2^ indicates that increased tHcy may be an epiphenomenon reflecting other mechanisms involved in atherothrombosis. Notably, results from the AtheroGene study demonstrated that tHcy was a markedly stronger predictor for cardiovascular risk in subjects with evidence of reduced antioxidative capacity.^32^ Findings from the present work suggest that tHcy is a particularly strong predictor of atherothrombosis in subjects with concomitant activation of the cellular immune response.

There is evidence that immune activation may deplete B‐vitamins by formation of reactive oxygen species.^33^ IFN‐γ released from Th1‐cells is considered the most important inducer of reactive oxygen species generation in macrophages,^15^ and IFN‐γ is highly expressed within atherosclerotic lesions.^34^ Production of neopterin parallels IFN‐γ‐mediated endogenous reactive oxygen species formation,^9^ and plasma neopterin is inversely correlated with systemic antioxidant levels.^35^ As recently demonstrated by Wu and coworkers,^36^ vascular oxidant stress strongly stimulates T‐cell proliferation and production of IFN‐γ, suggesting a bidirectional relationship between redox status impairment and the cellular immune response.

Regulation of the methionine–homocysteine cycle is strongly influenced by redox status.^37^ Notably, intracellular 5‐methyltetrahydrofolate^38^ and vitamin B12,^39^ both essential cofactors in the remethylation of homocysteine by methionine synthase, are highly susceptible to inactivation by oxidation. Taken together, increased tHcy in subjects with high neopterin levels may reflect a disturbed redox balance, considered to be deeply involved in atherogenesis and atherothrombosis. Also, homocysteine accumulates in parallel with neopterin upon stimulation of human peripheral blood mononuclear cells,^40^ indicating an additional direct link between immune activation and elevated tHcy. This finding is further substantiated by recent observations in sepsis patients, in whom tHcy increased only in nonsurvivors as compared with survivors.^41^

### Strengths and Limitations

Major strengths of the present study include the prospective design with long follow‐up, the large sample sizes, the detailed data on clinical baseline characteristics, and the replication of the results in a separate population. Also, both tHcy and neopterin have sufficient within‐person reproducibility to allow assessment by single measurements, which reduces the risk of regression dilution bias.^42^ As the collection of end points was based on patient administrative data, we cannot exclude the possibility of some misclassification or underreporting of clinical outcomes. However, we find it unlikely that any misclassifications differ according to tHcy or neopterin concentrations. Like any observational study, the possibility of residual confounding cannot be ruled out. Lastly, temporal changes in the management of patients with coronary heart disease may limit the generalizability of our results.

## Conclusion

In 2 independent cohorts of patients with suspected or verified CHD, the positive association between increased tHcy and risk of AMI was confined to patients with concomitantly elevated plasma neopterin. Our results motivate further research on the relationship between homocysteine metabolism, cellular immune activation, and atherothrombosis.

## Disclosures

None.

## Supporting information


**Table S1.** Association Between Plasma tHcy and Acute Myocardial Infarction According to Plasma Neopterin Among Participants (n=3346) Receiving Statin Therapy
**Table S2.** Association Between Plasma tHcy and Acute Myocardial Infarction According to Plasma Neopterin Among Participants (n=819) Not Receiving Statin Therapy
**Table S3.** Association Between Plasma tHcy and Acute Myocardial Infarction According to Plasma Neopterin Among Participants (n=2486) Without Previous Acute Myocardial Infarction
**Table S4.** Association Between Plasma tHcy and Acute Myocardial Infarction According to Plasma Neopterin Among Participants (n=1679) With Previous Acute Myocardial Infarction
**Table S5.** Baseline Characteristics Among Participants of the Norwegian Vitamin Trial (N=3749) According to Quartiles (n=937) of Plasma Total Homocysteine
**Figure S1.** Kaplan–Meier event‐free survival curves for patients with plasma homocysteine in quartiles 1 to 4. AMI indicates acute myocardial infarction; tHcy, total homocysteine.
