# Supplementary material for: Neopterin as an Effect Modifier of the Cardiovascular Risk Predicted by Total Homocysteine: A Prospective 2‐Cohort Study
Source: J Am Heart Assoc. 2017 Nov 2;6(11):e006500. doi: 10.1161/JAHA.117.006500 (PMC5721748; doi:10.1161/JAHA.117.006500)

# Supplemental Material

**Table S1.** The Association Between Plasma tHcy and Acute Myocardial Infarction According to Plasma Neopterin Among Participants (n=3346) Receiving Statin Therapy

| | Plasma neopterin | | | | |
|---|---|---|---|---|---|
| | ≤median* | | >median* | | |
| | HR† | 95% CI | HR† | 95% CI | $P_{int}$ |
| Unadjusted | 1.01 | (0.87-1.16) | 1.35 | (1.23-1.49) | 0.001 |
| Model 1‡ | 0.99 | (0.85-1.15) | 1.30 | (1.17-1.45) | 0.001 |
| Model 2§ | 0.96 | (0.82-1.12) | 1.25 | (1.12-1.40) | 0.002 |

*Median: 8.2 nmol/L.

†Per 1 SD increase in log transformed concentrations.

‡Adjusted for age and sex.

§Adjusted for age, sex, diabetes mellitus, current smoking, hypertension, apolipoprotein A1, and apolipoprotein B.

Abbreviations: HR: hazard ratio; SD: standard deviation; CI: confidence interval.

**Table S2.** The Association Between Plasma tHcy and Acute Myocardial Infarction According to Plasma Neopterin Among Participants (n=819) not Receiving Statin Therapy

| | Plasma neopterin | | | | |
|---|---|---|---|---|---|
| | ≤median* | | >median* | | |
| | HR† | 95% CI | HR† | 95% CI | $P_{int}$ |
| Unadjusted | 1.02 | (0.70-1.48) | 1.48 | (1.21-1.82) | 0.079 |
| Model 1‡ | 0.72 | (0.45-1.14) | 1.31 | (1.03-1.66) | 0.030 |
| Model 2§ | 0.71 | (0.44-1.15) | 1.28 | (1.00-1.63) | 0.044 |

*Median: 8.2 nmol/L.

†Per 1 SD increase in log transformed concentrations.

‡Adjusted for age and sex.

§Adjusted for age, sex, diabetes mellitus, current smoking, hypertension, apolipoprotein A1, and apolipoprotein B.

Abbreviations: HR: hazard ratio; SD: standard deviation; CI: confidence interval.

**Table S3.** The Association Between Plasma tHcy and Acute Myocardial Infarction According to Plasma Neopterin Among Participants (n=2486) Without Previous Acute Myocardial Infarction

| | Plasma neopterin | | | | |
|---|---|---|---|---|---|
| | ≤median* | | >median* | | |
| | HR† | 95% CI | HR† | 95% CI | $P_{int}$ |
| Unadjusted | 0.99 | (0.79-1.25) | 1.49 | (1.30-1.71) | 0.003 |
| Model 1‡ | 0.93 | (0.73-1.19) | 1.42 | (1.22-1.65) | 0.002 |
| Model 2§ | 0.88 | (0.68-1.13) | 1.36 | (1.16-1.60) | 0.002 |

*Median: 8.2 nmol/L.

†Per 1 SD increase in log transformed concentrations.

‡Adjusted for age and sex.

§Adjusted for age, sex, diabetes mellitus, current smoking, hypertension, apolipoprotein A1, and apolipoprotein B.

Abbreviations: HR: hazard ratio; SD: standard deviation; CI: confidence interval.

**Table S4.** The Association Between Plasma tHcy and Acute Myocardial Infarction According to Plasma Neopterin Among Participants (n=1679) with Previous Acute Myocardial Infarction

| | Plasma neopterin | | | | |
|---|---|---|---|---|---|
| | ≤median* | | >median* | | |
| | HR† | 95% CI | HR† | 95% CI | $P_{int}$ |
| Unadjusted | 0.96 | (0.81-1.14) | 1.23 | (1.10-1.37) | 0.023 |
| Model 1‡ | 0.95 | (0.80-1.14) | 1.17 | (1.03-1.32) | 0.017 |
| Model 2§ | 0.95 | (0.79-1.14) | 1.13 | (0.99-1.28) | 0.048 |

*Median: 8.2 nmol/L.

†Per 1 SD increase in log transformed concentrations.

‡Adjusted for age and sex.

§Adjusted for age, sex, diabetes mellitus, current smoking, hypertension, apolipoprotein A1, and apolipoprotein B.

Abbreviations: HR: hazard ratio; SD: standard deviation; CI: confidence interval.

**Table S5.** Baseline Characteristics Among Participants of the Norwegian Vitamin Trial (N=3749) According to Quartiles (n=937) of Plasma Total Homocysteine

| | **Quartiles of Plasma tHcy** | | | | |
|---|---|---|---|---|---|
| | First | Second | Third | Fourth | $P_{trend}$ |
| Plasma tHcy, µmol/L | 8.9 (8.0-9.5) | 11.1 (10.6-11.6) | 13.2 (12.6-13.9) | 17.6 (15.9-20.4) | … |
| Male sex, n (%) | 640 (68.6) | 695 (74.4) | 720 (77.0) | 706 (75.6) | <0.001 |
| Age, years | 58 (52-68) | 61 (53-70) | 65 (56-73) | 71 (59-77) | <0.001 |
| Plasma neopterin, nmol/L | 7.7 (6.1-10.1) | 8.1 (6.4-10.3) | 9.0 (7.0-11.7) | 11.0 (8.0-15.5) | <0.001 |
| Current smoking, n (%) | 449 (48.1) | 455 (48.8) | 439 (47.1) | 444 (47.6) | 0.665 |
| Diabetes mellitus, n (%) | 112 (12.1) | 84 (9.0) | 77 (8.3) | 91 (9.8) | 0.09 |
| BMI, kg/m$^2$ | 26.2 (23.9-28.7) | 26.0 (24.0-28.4) | 26.0 (24.0-28.4) | 25.5 (23.7-28.0) | <0.001 |
| Hypertension, n (%) | 231 (24.9) | 231 (25.0) | 264 (28.6) | 343 (37.3) | <0.001 |
| Previous PCI, n (%) | 48 (5.1) | 43 (4.6) | 41 (4.4) | 48 (5.1) | 0.949 |
| Previous MI, n (%) | 136 (14.6) | 135 (14.5) | 158 (16.9) | 245 (26.3) | <0.001 |
| eGFR, mL/min per 1.73m$^2$ | 82 (71-93) | 78 (67-90) | 75 (64-87) | 67 (54-82) | <0.001 |

Continuous variables are presented as medians (25$^{th}$-75$^{th}$ percentiles) and categorical variables as numbers (percentages).

Abbreviations: tHcy: total homocysteine; BMI: body mass index; PCI: percutaneous coronary intervention; MI: myocardial infarction; eGFR: estimated glomerular filtration rate.

**Figure S1.** Kaplan-Meier event-free survival curves for patients with plasma homocysteine in quartiles 1 to 4. AMI indicates acute myocardial infarction; and tHcy, total homocysteine.

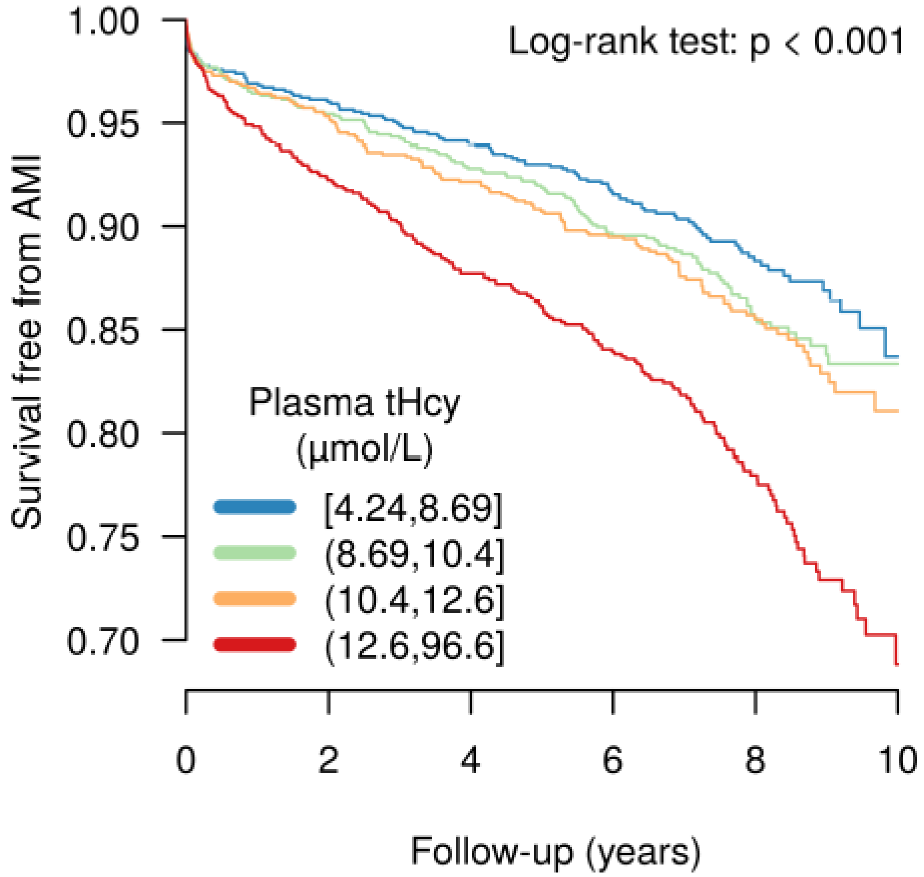

Supplement: Supplementary file 1 — Table S1. Association Between Plasma tHcy and Acute Myocardial Infarction According to Plasma Neopterin Among Participants (n=3346) Receiving Statin Therapy Table S2. Association Between Plasma tHcy and Acute Myocardial Infarction According to Plasma Neopterin Among Participants (n=819) Not Receiving Statin Therapy Table S3. Association Between Plasma tHcy and Acute Myocardial Infarction According to Plasma Neopterin Among Participants (n=2486) Without Previous Acute Myocardial Infarction Table S4. Association Between Plasma tHcy and Acute Myocardial Infarction According to Plasma Neopterin Among Participants (n=1679) With Previous Acute Myocardial Infarction Table S5. Baseline Characteristics Among Participants of the Norwegian Vitamin Trial (N=3749) According to Quartiles (n=937) of Plasma Total Homocysteine Figure S1. Kaplan–Meier event‐free survival curves for patients with plasma homocysteine in quartiles 1 to 4. AMI indicates acute myocardial infarction; tHcy, total homocysteine. [file JAH3-6-e006500-s001.pdf]
